# Emotion Reactivity Moderates the Association Between Momentary Negative Affect and Suicidal Thinking

**DOI:** 10.1002/brb3.71441

**Published:** 2026-04-28

**Authors:** Ellen M. Wittler, Kate H. Bentley, Matthew K. Nock, Evan M. Kleiman

**Affiliations:** ^1^ Department of Psychology Rutgers, The State University of New Jersey Piscataway New Jersey USA; ^2^ Department of Psychiatry Massachusetts General Hospital, Harvard Medical School Boston Massachusetts USA; ^3^ Department of Psychology Harvard University Cambridge Massachusetts USA

**Keywords:** EMA, emotion reactivity, negative affect, suicide

## Abstract

**Background:**

Most theories suggest suicidal thinking results from negative affect, but surprisingly few studies have assessed factors that may modify the relationship between negative affect and suicidal intent. Emotion reactivity is an understudied element of affective experience that may affect this relationship.

**Methods:**

This study examined ecological momentary assessment (EMA) data from two samples (*n =* 43 college students reporting suicidal ideation; *n* = 52 individuals with a history of suicide attempt recruited online) to investigate if trait emotion reactivity moderates the prediction of suicidal intent by negative affect. We hypothesized that increased trait emotion reactivity would strengthen the association between negative affect and next‐timepoint suicidal intent.

**Results:**

As hypothesized, across both samples, results indicated the association between momentary negative affect and next‐timepoint suicidal intent was stronger among those with greater trait emotion reactivity. Different facets of emotion reactivity appeared to drive these associations between studies (intensity in Study 1, and persistence and sensitivity in Study 2).

**Conclusion:**

These findings highlight the importance of including emotion reactivity in models of suicide and suggests trait‐level patterns of emotional responses are important to consider when modeling associations with momentary suicidal thoughts and behaviors. Between‐sample inconsistencies regarding which facets drove this relationship may speak to differences in emotional processing, which could hold important treatment implications.

## Graphical abstract

This study demonstrates that self‐reported emotion reactivity at baseline moderates the association between current negative affect and next‐timepoint suicidal intent in EMA data. Results are replicated across two samples (Study 1—college students reporting suicidal ideation; Study 2—adults with a history of suicide attempt).

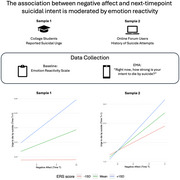

## 1 Introduction

Emotion reactivity is a trait‐level factor that contextualizes an individual's affective response following exposure to an emotional stimulus, particularly in the context of negative affect (Nock et al. 2008). Emotion reactivity consists of three facets: intensity—the strength of an individual's emotional response; persistence—the duration of an individual's emotional experience following exposure to an emotionally valenced stimulus; and sensitivity—an individual's tendency to respond to a wide array of stimuli (Nock et al. 2008). By this definition, we would expect someone with high emotion reactivity to have experienced repeated instances of extreme negative affect over the course of their life. Unsurprisingly, emotion reactivity has been associated with suicidal thoughts and behaviors ranging from thoughts of death and dying to making specific suicide plans (Chesin and Cascardi 2019; Liu et al. 2020; Nock et al. 2008; Shapero et al. 2019). In cross‐sectional studies, emotion reactivity has correlated specifically with current suicidal ideation (SI) and risk; however, it is not typically associated with long‐term history of suicidality (Chesin and Cascardi 2019; Nock et al. 2008; Tupler et al. 2015). Taken together, these findings paint a complicated picture of the relationship between emotion reactivity and suicide. Despite being a trait‐level variable, it seems trait emotion reactivity may better predict suicidality on a momentary basis, and hold less accuracy in predicting suicidality over time.

Elevated emotion reactivity implies someone generally has more intense, frequent, and longer experiences of negative affect. Importantly, longitudinal assessments of suicidality have indicated that long‐term experiences of negative affect are a risk factor for experiencing suicidal thoughts and behaviors (Lawson et al. 2022). Thus, emotion reactivity may not only describe how someone is going to respond emotionally; it also describes how someone has historically experienced emotions. Thus, we would expect someone with elevated emotion reactivity to be particularly vulnerable when experiencing momentary negative emotion.

Although emotion reactivity features momentary emotional experiences, prior research has primarily investigated emotion reactivity in cross‐sectional and correlational study designs (Chesin and Cascardi 2019; Dour et al. 2011; Kleiman, Ammerman, et al. 2014; Liu et al. 2020; Nock et al. 2008; Polanco‐Roman et al. 2018; Shapero et al. 2019; Wu et al. 2023). Previous research has also identified that momentary changes in negative affect (which would be expected with high emotion reactivity) are associated with SI (Liu et al. 2020; Armey et al. 2020). Still, no study has included trait emotion reactivity in investigations of momentary fluctuations in affective states and suicidal intent.

Emotion reactivity is a trait that reflects the tendency to experience large and acute fluctuations in affective states (particularly negative affective states). Though every person experiences negative affect, people vary in terms of how frequently and intensely they experience negative affect; these features are captured by emotion reactivity. The model of emotion reactivity described in foundational literature suggests those with elevated reactivity will experience negative affect more frequently, more intensely, and for longer periods of time (Nock et al. 2008). These repeated experiences of intense negative affect experienced by people with higher emotion reactivity may culminate in increased vulnerability for experiencing suicidal thoughts and behaviors (Lawson et al. 2022). While previous research has shown that fluctuations in negative affect are associated with SI (Kleiman et al. 2017), our ability to identify individuals at risk of making a suicide attempt remains low. Indeed, suicidal intent remains a high‐risk variable that is difficult to predict; an individual with high intent to die may require urgent intervention to prevent a suicide death. Examining the emotion reactivity trait may improve our understanding of who is at the highest risk of making a suicide attempt. Emotion reactivity may confer higher sensitivity and risk toward experiencing suicidal intent, particularly in an instance of intense negative emotion.

Momentary change in psychological states can be measured using ecological momentary assessment (EMA), a research method that collects data in real time (Ballard et al. 2021). EMA has been used to study different aspects of suicidality and to better understand how psychological states unfold in real time, including suicidal thoughts and behaviors (Armey et al. 2020; Kleiman et al. 2017; Sedano‐Capdevila et al. 2021). However, to our knowledge, no study examining suicidal thoughts or intent has included trait‐level emotion reactivity in their analyses. Emotion reactivity is associated with suicidal thoughts cross‐sectionally and could signal a long‐term pattern of intense negative affect. Thus, emotion reactivity may moderate the relationship between momentary negative affect and suicidal intent, such that those with higher emotion reactivity are more likely to experience elevated suicidal intent when experiencing negative affect.

While previous research has found that SI and negative affect correlate, predicting next‐timepoint suicidality remains a challenge (Kleiman et al. 2017). Recently, an EMA study examining the prediction of next‐timepoint active and passive suicidal thoughts found that neither affect nor overall emotional distress predicted next‐timepoint active suicidal thoughts (Kleiman et al. 2026). This same study found that only sadness predicted next‐timepoint passive suicidal thoughts, though passive thoughts are typically less acute in terms of need for immediate intervention. However, suicidal thinking is highly heterogeneous, and particularly for those with higher emotion reactivity, negative emotion may predict active SI, such as suicidal intent. As explained previously, those with elevated emotion reactivity may be at higher risk for suicide attempt via a long‐term history of intense negative affect. Additionally, heightened emotion reactivity is characteristic in high‐impulsivity presentations, like borderline personality disorder (BPD) (Nock et al. 2008). Previous research suggests that in high‐impulsivity presentations like BPD, negative emotions can amplify negative thinking patterns over the course of hours, and predict future impulsive behaviors, like self‐harm and suicidal behaviors (Selby et al. 2016). Emotion reactivity may be an important piece of the puzzle for predicting active SI, particularly suicidal intent, that has been neglected in previous models of suicidality prediction.

This study seeks to investigate the impact trait‐level emotion reactivity has on the association between momentary negative affect and next‐timepoint suicidal intent. Those with higher emotion reactivity have likely experienced elevated negative affect over long periods of time, and this is a risk factor for suicidal thoughts and behaviors. Thus, we would expect that individuals with elevated emotion reactivity would experience greater suicidal intent following experiences of negative affect. Including emotion reactivity in our model, particularly interacting with negative affect, better reflects the differences in emotional processing that are hypothesized to occur with elevated emotion reactivity, a common feature among suicidal individuals (Chesin and Cascardi 2019; Liu et al. 2020; Nock et al. 2008; Shapero et al. 2019). In addition to this primary investigation we evaluated several exploratory models examining the different facets of emotion reactivity (persistence, sensitivity, intensity) to identify if an underling component of the trait could be driving any observed relationship we find.

## 2 Materials and Method

### 2.1 Participants

#### 2.1.1 Study 1: College Intervention Study

Participants in Study 1 included 43 college students selected from a larger registered clinical trial of a brief emotion management skills‐based intervention (NCT04636151) (Kleiman et al. 2022), involving 177 participants. Inclusion in the present analyses required at least one instance of reporting nonzero suicidal urges during the study.

#### 2.1.2 Study 2: Community Sample With Previous Suicide Attempt

Participants in Study 2 were 52 adults recruited online from various suicide‐related communities selected from a larger study of 54 adults (Kleiman et al. 2017). The two participants who were not included in this manuscript did not have complete baseline data.

### 2.2 Procedure

#### 2.2.1 Study 1: College Intervention Study

All procedures for Study 1 were approved by the Rutgers Institutional Review Board (IRB ID: Pro2020002347). Participants in Study 1 were recruited via emails distributed to student listservs that advertised a workshop for emotion management. Those who were interested completed a consent form and brief set of baseline measures, then attended a 90‐min in‐person or virtual workshop where they learned emotion management skills. After the workshop, they completed up to 8 weeks of a 4× daily smartphone‐based EMA via the MetricWire app.

To ensure participant safety, study staff were notified immediately whenever participants reported elevated suicide intent via EMA (e.g., reporting 8/10 or greater on the item “What is your current intent to die by suicide?”). When a risk notification was sent, study staff promptly reviewed the data and, if needed, conducted a more in‐depth suicide risk assessment. Participants who were determined to be at moderate risk of suicide attempt were directed to an appropriate support resource (e.g., speaking with the on‐call therapist at the student counseling service). In cases of determined imminent risk, study staff contacted an urgent support resource, such as the student counseling center or the on‐campus psychiatric hospital, to provide direct outreach to the participant.

#### 2.2.2 Study 2: Community Sample With Previous Suicide Attempt

All procedures for Study 2 were approved by the Harvard Institutional Review Board (IRB ID: 15–1975). Participants in Study 2 were recruited via posts to suicide‐related online communities. Those who were interested completed a screener, consent, and a short set of baseline surveys, followed by a 4× daily smartphone‐based EMA via the mEMA app for 28 days. There was no restriction on the country of residence for participants in this study.

To ensure participant safety, all participants were provided resources for support in the event of experiencing intensifying SI. Because online communities can include individuals from across the globe, international resources were included in the information provided to participants. We did not conduct real‐time review of data in this study because real‐time data review was not technologically possible when these data were collected (2016). Participants were made aware in the consent form that data were not reviewed in real time.

### 2.3 Measures

We used the same measures across both studies.

#### 2.3.1 Baseline Measures

Emotion reactivity was assessed in both studies at baseline using the Emotion Reactivity Scale (ERS) (Nock et al. 2008), a 21‐item measure of trait emotion reactivity. In this scale, higher scores indicate greater levels of emotion reactivity. The ERS includes three subscales for each of the three facets of emotion reactivity (persistence, intensity, sensitivity) as well as a full‐scale composite score.

#### 2.3.2 EMA Measures

Momentary negative affect was assessed using a four‐item composite score including affect items assessed across both studies: agitated, angry, burdensome, and hopeless. All affect items were the same across both studies (e.g., “Right now, how hopeless do you feel”). In Study 1, participants rated their responses using a 0–10 scale, where 0 meant “not at all” and 10 meant “very much.” In Study 2, participants rated their responses using a 0–4 scale, where 0 meant “not at all” and 10 meant “very much.”

Suicide intent was assessed using single‐item scales in both studies. In Study 1, participants were asked to respond to the question “Right now, how strong is your intention to kill yourself?,” using a 0–10 scale, where 0 meant “not at all” and 10 meant “very much.” In Study 2, participants were asked to respond to the question “How strong is your intention to kill yourself right now?,” using a 0–4 scale, where 0 meant “not at all” and 10 meant “very intense.”

### 2.4 Analytic Strategy

We followed the same analytic strategy across both studies.

#### 2.4.1 Multilevel Confirmatory Factor Analysis (CFA)

To justify the use of the four affect labels (agitated, angry, burdensome, and hopeless) in a singular consolidated “negative affect” construct, we conducted a multilevel CFA in the lavaan R package for each study (Rosseel 2012). A multilevel CFA was chosen to assess reliability of the composite negative affect variable while appropriately accounting for the nested structure of the data in these studies. For each CFA, the model specified a single latent variable (negative affect) at both the within‐person and between‐person levels. At each level, the four negative affect items assessed in EMA data collection were specified as indicator variables (agitated, angry, burdensome, and hopeless). Both models were estimated using maximum likelihood.

#### 2.4.2 Multilevel Moderated Modeling

We estimated a series of multilevel moderated models testing whether trait emotion reactivity moderated any association between momentary negative affect and next‐timepoint suicidal intent. We were also interested in exploring specific associations for each subscale of the ERS. Thus, four model assessments were conducted for each study: one for the total ERS score and three additional for each ERS subscale. In all models, which were estimated using the lme4 package (Bates et al. 2015), the predictor was composite negative affect, person‐mean centered, and measured at time T; the moderator was the relevant emotion reactivity measure (full ERS a subscale), grand mean centered, and measured at the person level; and the outcome was suicidal urge at time T + 1. All models specified fixed slopes and random intercepts, and utilized the restricted maximum likelihood (REML) estimation method. In instances where the cross‐level interaction between negative affect and emotion reactivity was significant, we further plotted (using the sjPlot package) (Lüdecke 2022) and probed (using the interactions package) (Long 2019) the interaction.

## 3 Results

### 3.1 Descriptives

Descriptive statistics for all study measures are presented in Table 1. Means across all variables of interest were lower in the college intervention study than in the online study of previous suicide attempters.

**TABLE 1:** Sample‐level descriptive statistics for all study variables.

	Study 1				Study 2			
	Mean	SD	*α*	ICC	Mean	SD	*α*	ICC
**Person‐level measures**								
ERS—total score	5.65	2.72	0.95	—	9.92	2.18	0.93	—
ERS—persistence	1.92	0.98	0.78	—	3.48	0.76	0.75	—
ERS—sensitivity	1.72	0.94	0.90	—	3.12	0.86	0.87	—
ERS—intensity	2.01	1.03	0.89	—	3.32	0.83	0.90	—
								
**EMA measures**								
Negative affect	1.74	1.84	0.76	0.60	2.49	1.17	0.88	0.60
Urge to die by suicide	0.15	0.75	—	0.53	2.23	1.36	—	0.55

*Note*: Alpha for EMA measures is between‐person reliability.

Abbreviation: ICC = intraclass correlation.

### 3.2 Study 1: College Intervention Study

#### 3.2.1 Demographics

The average age of participants in Study 1 was 24.01 years (SD *=* 5.37, range = 18.71–44.10 years). Of the sample, 69.77% identified as cisgender female, 20.9% identified as cisgender male, and the remainder identified as nonbinary or gender nonconforming. The sample was 34.9% white, 32.6% Asian, 11.6% Black/African American, and the remainder endorsed multiple or other races. The sample included 16.3% Hispanic/Latinx.

#### 3.2.2 Multilevel CFA

Overall, the multilevel CFA demonstrated acceptable model fit (comparative fit index [CFI] = 0.96). Root mean square error of approximation was slightly lower than ideal (RMSEA = 0.08), but this is typical in datasets with large sample sizes. At the within‐person level, model fit was good (SRMR = 0.03), and all indicators loaded significantly onto the latent negative affect factor (standardized loadings = 0.54–0.65, *p*‐values < 0.001). At the between‐person level, model fit was good (SRMR = 0.03), and all indicators loaded significantly onto the latent negative affect factor (standardized loadings = 0.72–0.84, *p*‐values < 0.001, see Figure S1), suggesting a justified negative affect construct across individuals. Full factor loading is available in Figure S1.

Reliability was assessed using McDonald's *ω*, and was calculated separately at the within‐ and between‐person levels using estimated factor loadings and residual variances. At the within‐person level, reliability was acceptable (*ω* = 0.695), indicating appropriate internal consistency of momentary affect ratings for the individual. At the between‐person level, reliability was also acceptable (*ω* = 0.871), reflecting moderately high consistency across the sample.

The purpose of this CFA was to justify the use of a composite negative affect score in subsequent analyses. Fit indices suggested appropriate model fit, and factor loading appears consistent and substantial across levels. These results suggest the composite negative affect measure is suitable to use for the primary analyses in this dataset.

#### 3.2.3 Results

Results of the multilevel moderated models for each of the ERS scales and total score for Study 1 are presented in Table 2. There were significant affect X ERS interactions for the total ERS score and for the intensity subscale, such that higher composite negative affect was associated with higher ERS—total and higher ERS—intensity.

**TABLE 2:** Baseline ERS subscale X negative affect (time T) predicting urge to die by suicide at time T + 1 (Study 1: college intervention study).

	Total ERS			Persistence subscale			Sensitivity subscale			Intensity subscale		
	*B*	95% CI	*p*	*B*	95% CI	*p*	*B*	95% CI	*p*	*B*	95% CI	*p*
Intercept	0.23	0.00–0.45	0.050	−0.22	−0.78 to 0.35	0.456	−0.13	−0.60 to 0.34	0.584	−0.10	−0.61 to 0.42	0.714
Urge to die (T)	0.25	0.21–0.28	**< 0.001**	0.25	0.22–0.29	**< 0.001**	0.25	0.22–0.29	**< 0.001**	0.25	0.22–0.29	**< 0.001**
Negative affect (T)	0.03	0.01–0.05	**0.002**	< 0.001	−0.03 to 0.02	0.856	< 0.001	−0.02 to 0.02	0.921	−0.01	−0.03 to 0.01	0.158
ERS subscale	0.08	−0.01 to 0.17	0.322	0.21	−0.03 to 0.46	0.089	0.22	−0.03 to 0.47	0.080	0.17	−0.07 to 0.40	0.160
Affect X ERS subscale	0.01	0.00–0.01	**0.007**	0.01	−0.00 to 0.02	0.180	0.01	−0.00 to 0.03	0.156	0.02	0.01–0.03	**0.001**

#### 3.2.4 Primary Findings

**FIGURE 1:**
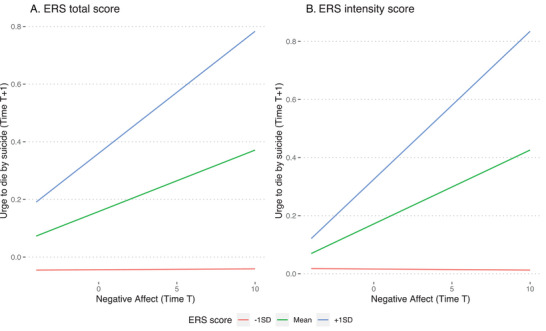
Interaction plots for the significant interactions in Study 1 (college intervention study).

The interaction between ERS—total score and negative affect at time T, predicting urge to die by suicide at T + 1, is plotted in Figure 1A. Simple slopes probes revealed that the slopes for the relationship between baseline negative affect at T and urge to die by suicide at T + 1 were significant at high (+1SD, *b* = 0.042, 95% CI = [0.001, 0.170], *p* < 0.001) and mean (*b* = 0.021, 95% CI = [0.005, 0.038], *p* = 0.007) levels of ERS, but not significant at low levels of ERS (*b* < 0.001, 95% CI = [−0.020, 0.021], *p* = 0.475).

#### 3.2.5 Exploratory Analyses of Subscales

The interaction between ERS—intensity score and negative affect at time T, predicting urge to die by suicide at T + 1, is plotted in Figure 1B. Simple slopes probes revealed a similar pattern to the overall scale. Slopes for the relationship between baseline negative affect at T and urge to die by suicide at T + 1 were significant at high (+1SD, *b* = 0.051, 95% CI = [0.027, 0.076], *p* < 0.001) and mean (*b* = 0.025, 95% CI = [0.009, 0.042], *p* = 0.002), but not significant at low (*b* ← 0.001, 95% CI = [−0.019, 0.019], *p* = 0.490) levels of ERS—intensity.

### 3.3 Study 2: Community Sample With Previous Suicide Attempt

#### 3.3.1 Demographics

The average age of participants in Study 2 was 23.48 years (SD *=* 4.37, range = 18.1–36.8 years). Of the sample, 75% identified as cisgender female, 19.2% identified as cisgender male, and the remainder identified as nonbinary or gender nonconforming, or did not wish to report their gender. The sample was 73.1% white, 7.6% Asian, and the remainder endorsed another race. Of the sample, 7.6% identified as Hispanic/Latinx.

#### 3.3.2 Multilevel CFA

Overall, the multilevel CFA demonstrated acceptable model fit (CFI = 0.93). At the within‐person level, model fit was good (SRMR = 0.04), and all indicators loaded significantly onto the latent negative affect factor (standardized loadings = 0.52–0.70, *p*‐values < 0.001). At the between‐person level, factor loading was strong (standardized loadings = 0.71–0.90, *p*‐values < 0.001, see Figure S2), suggesting a justified negative affect construct across individuals; however, some fit indices suggested model misfit (RMSEA = 0.12; SRMR = 0.11).

Reliability was assessed using McDonald's *ω*, and was calculated separately at the within‐ and between‐person levels using estimated factor loadings and residual variances. At the within‐person level, reliability was acceptable (*ω* = 0.735), indicating moderate internal consistency of momentary affect ratings for the individual. At the between‐person level, reliability was also acceptable (*ω* = 0.870), reflecting moderately high consistency across the sample.

The purpose of this CFA was to justify the use of a composite negative affect score in subsequent analyses. While some fit indices suggest model misfit, factor loadings appear consistent and substantial across levels. These results suggest the composite negative affect measure is suitable to use for the primary analyses in this dataset.

#### 3.3.3 Results

Results of the multilevel moderated models for each of the ERS scales and total score for Study 2 are presented in Table 3. There were significant affect X ERS interactions for the total ERS score and for the persistence and sensitivity subscales, such that higher composite negative affect was associated with higher ERS—total, higher ERS—persistence, and higher ERS—sensitivity.

**TABLE 3:** Baseline ERS subscale X negative affect (time T) predicting urge to die by suicide at time T + 1 (Study 2: suicide attempters recruited online).

	Total ERS			Persistence subscale			Sensitivity subscale			Intensity subscale		
	*B*	95% CI	*p*	*B*	95% CI	*p*	*B*	95% CI	*p*	*B*	95% CI	*p*
Intercept	2.09	1.84–2.35	**< 0.001**	2.08	1.83–2.33	**< 0.001**	2.09	1.84–2.35	**< 0.001**	2.07	1.82–2.32	**< 0.001**
Urge to die (T)	0.33	0.28–0.38	**< 0.001**	0.33	0.28–0.38	**< 0.001**	0.33	0.28–0.38	**< 0.001**	0.33	0.28–0.38	**< 0.001**
Negative affect (T)	0.16	0.10–0.22	**< 0.001**	0.15	0.08–0.21	**< 0.001**	0.17	0.10–0.23	**< 0.001**	0.16	0.09–0.22	**< 0.001**
ERS subscale	0.06	−0.06 to 0.18	0.322	0.25	−0.08 to 0.58	0.138	0.03	−0.27 to 0.33	0.867	0.19	−0.12 to 0.50	0.237
Affect X ERS subscale	0.03	0.01–0.05	**0.011**	0.10	0.03–0.18	**0.004**	0.07	0.02–0.13	**0.011**	0.05	−0.01 to 0.11	0.134

#### 3.3.4 Primary Findings

**FIGURE 2:**
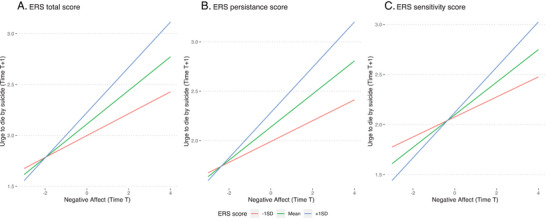
Interaction plots for the significant interactions in Study 2 (individuals who previously attempted suicide recruited online).

The interaction between ERS—total score and negative affect at time T, predicting urge to die by suicide at T + 1, is plotted in Figure 2A. Simple slopes probes revealed that the slopes for the relationship between baseline negative affect at T and urge to die by suicide at T + 1 were significant at high (+1SD, *b* = 0.22, 95% CI = [0.143, 0.302], *p* < 0.001), mean (*b* = 0.165, 95% CI = [0.103, 0.227], *p* < 0.001), and low levels of ERS (*b* = 0.108, 95% CI = [0.036, 0.180], *p* = 0.003).

#### 3.3.5 Exploratory Analyses of Subscales

The interaction between ERS—persistence score and negative affect at time T, predicting urge to die by suicide at T + 1, is plotted in Figure 2B. Simple slopes probes revealed a similar pattern to the overall scale. Slopes for the relationship between baseline negative affect at T and urge to die by suicide at T + 1 were significant at high (+1SD, *b* = 0.229, 95% CI = [0.15, 0.309], *p* < 0.001), mean (*b* = 0.168, 95% CI = [0.106, 0.23], *p* < 0.001), and low (*b* = 0.106, 95% CI = [0.036, 0.176], *p* = 0.003) levels of ERS—persistence.

The interaction between ERS—sensitivity score and negative affect at time T, predicting urge to die by suicide at T + 1, is plotted in Figure 2C. Simple slopes probes revealed a similar pattern to the overall scale. Slopes for the relationship between baseline negative affect at T and urge to die by suicide at T + 1 were significant at high (+1SD, *b* = 0.229, 95% CI = [0.15, 0.309], *p* < 0.001), mean (*b* = 0.032, 95% CI = [0.225, 5.16], *p* < 0.001), and low (−1SD*, b* = 0.042, 95% CI = [0.308, 5.405], *p* = 0.006) levels of ERS—sensitivity.

### 3.4 Summary of Findings Across Studies

Overall, we found consistency across both studies in the significance and direction of the interaction between ERS—total score and negative affect at time T predicting urges to die by suicide at T + 1. We identified a stronger association between negative affect and suicidal intent among individuals with higher reported emotion reactivity. When examining individual ERS subscales, there was less consistency across studies; although all significant interactions had the same general pattern (stronger association between negative affect and suicidal intent with higher reactivity), the specific subscales for which significant interaction emerged differed by study. In Study 1, only the intensity–negative affect interaction was significant. In Study 2, only the persistence–negative affect and sensitivity–negative affect interactions were significant.

## 4 Discussion

As expected, emotion reactivity moderated the association between negative affect and next‐timepoint suicidal intent across both studies. Specifically, the association between negative affect and next‐timepoint suicidal intent was stronger for those with elevated emotion reactivity. This finding suggests that individuals with higher emotion reactivity were more sensitive to the negative affect they experienced, and in turn that negative affect was more likely to predict suicidal urges in the near future. This finding was consistent across two different samples with different levels of severity (college students vs. clinical sample of adults). Overall, these findings indicate emotion reactivity is an important factor in real‐time experience of suicidal intent over time, potentially reflecting an underlying emotional vulnerability for suicidal intent. Furthermore, this supports the value of including trait‐level variables like emotion reactivity in models of real‐time negative affect.

The exploratory investigation of ERS subscales was surprising in that the observed associations were not consistent across both groups. In Study 1, only the intensity subscale significantly moderated negative affect and next‐timepoint suicidal intent; in Study 2, both the persistence and sensitivity subscales moderated negative affect and next‐timepoint suicidal intent, but not the intensity facet. The inconsistency between which facet drove the overall relationship may suggest several conclusions. All three subscales significantly moderated the relationship between negative affect and suicidal intent, but which subscale drove the effect differed between study samples. Given that our samples were recruited with different criteria (Study 1—college students; Study 2—previous suicide attempts), this may suggest that the three facets of emotion reactivity confer risk differently depending on other group‐level factors not controlled for in this study. This finding is similar to ideographic weakest link models of psychopathology in which an individual's risk is characterized by the strongest feature of a variety of vulnerability components (Kleiman, Riskind, et al. 2014). Weakest link models have previously been supported in suicide research and thus may be relevant in the current study. Applied in this context, this may mean that risk for suicide intent in our college student sample is being driven by the tendency to experience more intense affect, and in our previous suicide attempt sample, risk is being driven both by greater sensitivity and greater persistence of negative affect. If supported, this conclusion could suggest that different intervention strategies may be better suited for certain individuals, depending on what facet of emotion reactivity is primarily driving the relationship between negative emotion and suicidal intent. Perhaps, for example, those for whom distress is associated with elevated intensity would be well‐suited for distress tolerance interventions; for those with elevated duration, mindfulness would be effective; and those with elevated sensitivity may benefit from acceptance‐based principles. Regardless, further investigation is warranted to confirm these findings. The assessment of individual ERS facets was exploratory, and sample sizes were relatively small in both studies. Thus, replicating these model assessments in a larger sample would help increase confidence in these exploratory findings.

Additionally, these findings speak overall to the importance of examining the interactions between trait‐level variables and the momentary variables they are theorized to affect. The theory behind emotion reactivity suggests that people with elevated reactivity will experience more extreme (more intense, longer lasting, and more frequent) emotional events over time (Nock et al. 2008). While emotion reactivity has been assessed in previous studies of suicidal thoughts and behaviors, it has not been considered as a potential moderator when examining suicidality in EMA studies (Chesin and Cascardi 2019; Nock et al. 2008; Shapero et al. 2019). Emotion reactivity as a trait‐like construct fundamentally suggests that people's experiences of emotion are different (Nock et al. 2008), and previous research suggests those who have experienced more extreme negative affect over time are more vulnerable to experiencing suicidal thoughts and behaviors (Lawson et al. 2022). Thus, evaluating the effects of emotion reactivity alongside EMA data was an appropriate application of the underlying model. Examining other trait‐level variables alongside momentary variables that may be impacted by traits will be important to better understand psychological traits and psychopathology in future research.

While this study and these findings bring important insights to this field of study, there are limitations that should be addressed in future research. Samples sizes were relatively small in both studies. Given the replication of our primary finding across both samples, we are confident in these results; however, repetition of these assessments in a larger sample is warranted to ensure sufficient power. Additionally, we relied on a single‐item assessment of suicidal intent. This was done to maintain consistency across both studies and precisely target suicidal intent; however, experiences with suicidal thoughts and behaviors are nuanced and we recognize that slight changes in assessment wording can impact participant response. Future studies may consider multi‐item assessments to encapsulate more potential variability in participant response.

Suicidal thoughts and behaviors occur across the spectrum of psychiatric severity levels, though both studies were limited in terms of subject severity. Study one included college students without any specific requirement for experiencing SI, though a large enough portion of study participants reported suicidal intent for these analyses to be appropriate. Study two targeted individuals who endorsed a past‐year suicide attempt. Across both studies, there were still relatively low levels of suicidal thinking; thus, it is important to replicate this study in an acute psychiatric sample to understand how this model applies to individuals with more severe symptoms. In future studies, demographic variables should be explored in greater depth to determine how emotion reactivity may systematically affect people differently. Previous research suggests that demographic factors like gender impact how emotion reactivity affects a person's behavior (Kleiman, Ammerman, et al. 2014). In a larger sample with greater representation across different groups, demographic variables should be investigated.

## 5 Conclusion

This research offers important strengths that have not been available in other studies of emotion reactivity. By including trait emotion reactivity as a moderating variable, we revealed that momentary experiences of negative affect may be predictive of next‐timepoint suicidal intent for those with elevated emotion reactivity. Additionally, our ability to replicate these findings across two different samples of varying severity highlights the consistency of this finding. Overall, this study brings to light why including trait‐level variable like emotion reactivity in models of suicidality is important for understanding relationships between momentary variables and suicidality.

## Author Contributions

**Ellen M. Wittler**: conceptualization, writing – original draft, formal analysis. **Evan M. Kleiman**: formal analysis, supervision, writing – review and editing, conceptualization, investigation. **Kate H. Bentley**: conceptualization, writing – review and editing. **Matthew K. Nock**: writing – review and editing, conceptualization, investigation.

## Funding

Funding for Study 1 (college student intervention study) was provided by the National Institute of Mental Health, grant R34MH113757. Funding for Study 2 (participants with previous suicide attempt recruited online) was provided by the Pershing Square Venture Fund for Research on the Foundations of Human Behavior.

## Ethics Statement

Ethical approval for Study 1 (college student intervention study) was provided by the Rutgers Institutional Review Board (IRB ID: Pro2020002347). Ethical approval for Study 2 (participants with previous suicide attempt recruited online) was granted by the Harvard Institutional Review Board (IRB ID: 15–1975).

## Conflicts of Interest

Matthew K. Nock receives publication royalties from Macmillan, Pearson, and UpToDate. He has been a paid consultant in the past 3 years for Cambridge Health Alliance, and for legal cases regarding a death by suicide. He has stock options in Cerebral Inc. He is an unpaid scientific advisor for Empatica, Koko, and TalkLife. Evan M. Kleiman reports a relationship with John Wiley & Sons Inc that includes consulting or advisory services. The other authors declare no conflicts of interest.

## Supporting information




**Supplementary Figure**: brb371441‐sup‐0001‐FigureS1.docx

**Supplementary Figure**: brb371441‐sup‐0002‐FigureS2.docx

## Data Availability

The datasets used for the current study are available from the corresponding author upon reasonable request.
