# Supplementary material for: Emotion Reactivity Moderates the Association Between Momentary Negative Affect and Suicidal Thinking
Source: Brain Behav. 2026 Apr 28;16(5):e71441. doi: 10.1002/brb3.71441 (PMC13125430; doi:10.1002/brb3.71441)
Supplement: Supplementary file 2 — Supplementary Figure: brb371441‐sup‐0002‐FigureS2.docx [file BRB3-16-e71441-s001.docx]

**Supplemental Figure 2. Negative affect CFA standardized loadings for Study 2 (individuals who previously attempted suicide recruited online)**

**
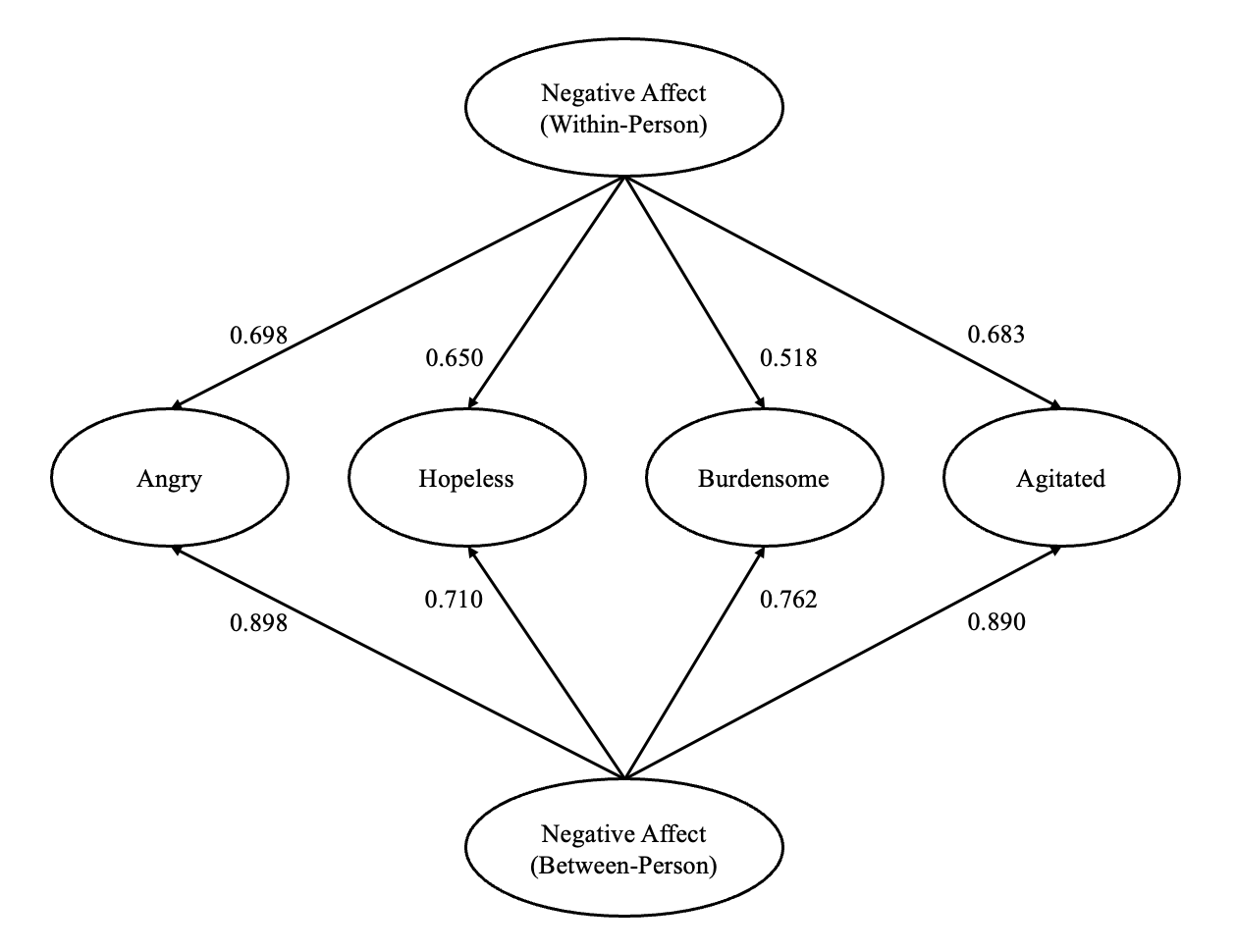
**
